# A Rapidly Enlarging Squamous Inclusion Cyst in an Axillary Lymph Node following Core Needle Biopsy

**DOI:** 10.1155/2012/418070

**Published:** 2012-04-17

**Authors:** Cunxian Zhang, Jinjun Xiong, M. Ruhul Quddus, Joyce J. Ou, Katrine Hansen, C. James Sung

**Affiliations:** Department of Pathology and Laboratory Medicine, Women & Infants Hospital of Rhode Island and Warren Alpert Medical School of Brown University, 101 Dudley Street, Providence, RI 02905, USA

## Abstract

A 73-year-old woman was found to have a 1.7 cm axillary mass, for which a core needle biopsy was performed. The specimen revealed fragmented squamous epithelium surrounded by lymphoid tissue consistent with a squamous inclusion cyst in a lymph node, but a metastatic squamous cell carcinoma could not be excluded. Within one month, the lesion enlarged to 5 cm and was excised. Touch preparation cytology during intraoperative consultation displayed numerous single and sheets of atypical epithelioid cells with enlarged nuclei and occasional mitoses, suggesting a carcinoma. However, multinucleated giant cells and neutrophils in the background indicated reactive changes. We interpreted the touch preparation as atypical and recommended conservative surgical management. Permanent sections revealed a ruptured squamous inclusion cyst in a lymph node with extensive reactive changes. Retrospectively, the atypical epithelioid cells on touch preparation corresponded to reactive histiocytes. This is the first case report of a rapidly enlarging ruptured squamous inclusion cyst in an axillary lymph node following core needle biopsy. Our case demonstrates the diagnostic challenges related to a ruptured squamous inclusion cyst and serves to inform the readers to consider this lesion in the differential diagnosis for similar situations.

## 1. Introduction

Benign epithelial inclusions in lymph nodes refer to nonneoplastic ectopic epithelium in lymph nodes. Various types are described in the literature, including tubal-like glands in pelvic lymph nodes [1], thyroid gland tissue in cervical lymph nodes [2], and mesothelium in mediastinal lymph nodes [3]. Epithelial inclusions in axillary lymph nodes are uncommon tumor-like lesions, with only about 40 cases reported in the literature [4–13]. They can display various histologic features. While most epithelial inclusions in axillary lymph nodes consist of glands alone or associated with cysts lined by apocrine or squamous epithelium [4, 8, 9, 14, 15], five others have been described as cysts lined by apparently pure stratified squamous epithelium with a prominent granular cell layer and hyperkeratosis [5–8, 10]. Epithelial inclusions may be incidental findings during procedures for other conditions [4–7], but they may also present as enlarged lymph nodes worrisome for malignancy [8–10]. We report a rapidly enlarging squamous inclusion cyst of the axillary lymph node following core needle biopsy, mimicking malignancy during pre- and intraoperative workups.

## 2. Case Presentation

**Figure 1:**
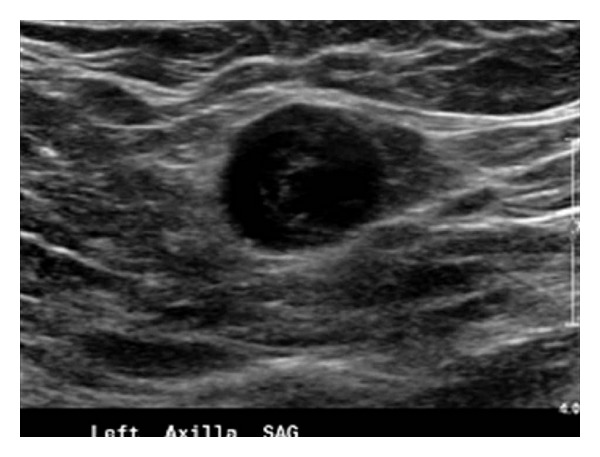
Ultrasound image at the time of core needle biopsy shows a well-circumscribed, 1.7 × 1.5 × 1.4 cm mass in the left axilla.

A 73-year-old woman presented for a regular checkup. Mammography was performed and showed a mass in the patient's left axilla. The mass measured 1.7 × 1.5 × 1.4 cm by ultrasound (Figure 1). The patient denied fever, fatigue, or weight loss. Her past medical history included excision of a left breast papilloma 3 years prior to the current presentation. She did not have any history of malignancy.

Core needle biopsy of the left axillary mass showed fragmented squamous epithelium surrounded by lymphoid tissue. The changes were consistent with a squamous inclusion cyst in a lymph node, but a metastatic squamous cell carcinoma could not be ruled out. Within one month following core needle biopsy, the lesion rapidly enlarged to 5 cm and become clinically alarming.

**Figure 2:**
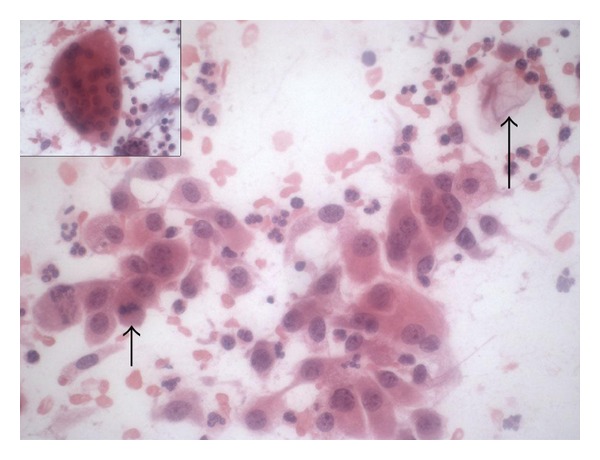
Intraoperative touch preparation slide exhibits single and clusters of atypical epithelioid cells with prominent nucleoli and focal mitotic figures (wide short arrow). The slide also shows keratin debris (narrow long arrow), numerous neutrophils, and occasional multinucleated foreign-body-type giant cells (insert). Hematoxylin and eosin stain; magnification: ×400.

The lesion was excised and sent for intraoperative pathology consultation. The specimen showed a mass-like lesion measuring 5 cm in greatest dimension. A touch preparation slide was made and showed numerous single and sheets of atypical epithelioid cells with enlarged nuclei, prominent nucleoli, and focal mitotic figures, suggesting a carcinoma (Figure 2). The background slide exhibited keratin debris, abundant neutrophils, and occasional multinucleated foreign-body-type giant cells, indicating reactive changes. We interpreted the touch preparation cytology as atypical and recommended conservative surgical management.

**Figure 3:**
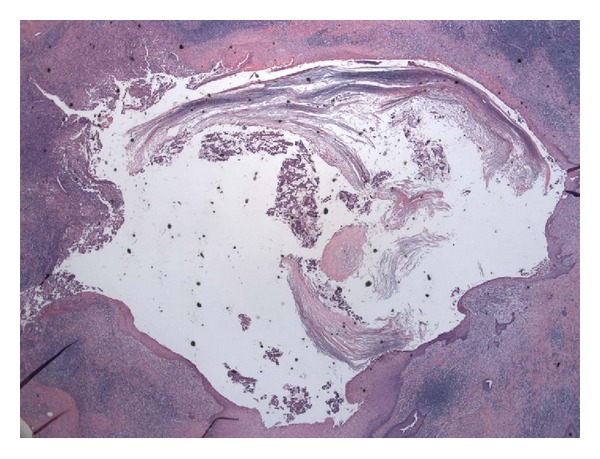
Low power view of permanent section shows a squamous inclusion cyst within a lymph node. Hematoxylin and eosin stain; magnification: ×40.

**Figure 4:**
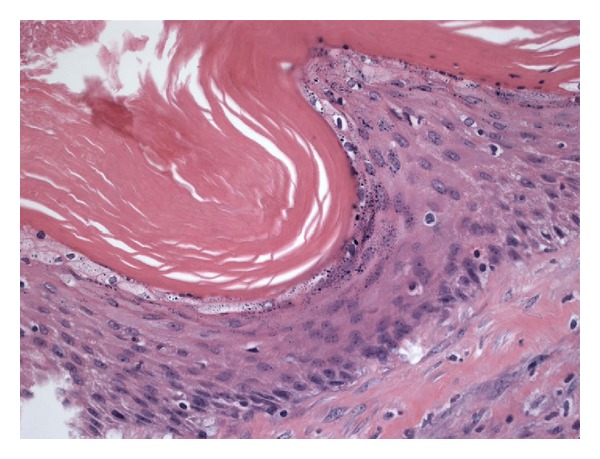
Parts of the cyst are lined by stratified squamous epithelial cells with thick laminated hyperkeratotic material. Hematoxylin and eosin stain; magnification: ×400.

**Figure 5:**
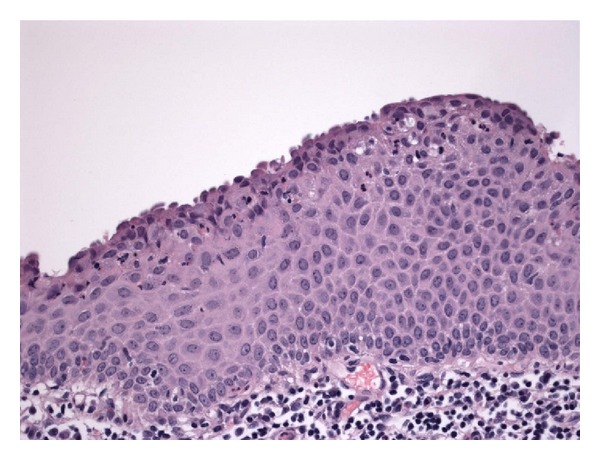
Some parts of the cyst are lined by multiple layers of polygonal squamous cells without a granular cell layer or hyperkeratosis. Hematoxylin and eosin stain; magnification: ×400.

**Figure 6:**
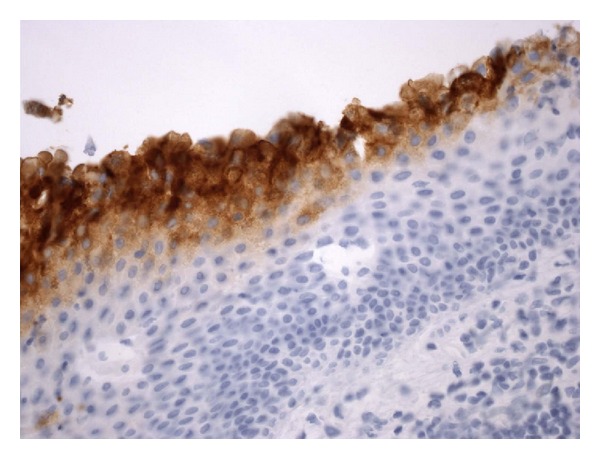
In the parts of the cyst showing multiple polygonal squamous cells without a granular cell layer or hyperkeratosis, the inner cyst cells are immunopositive for carcinoembryonic antigen. Immunostain; magnification: ×400.

**Figure 7:**
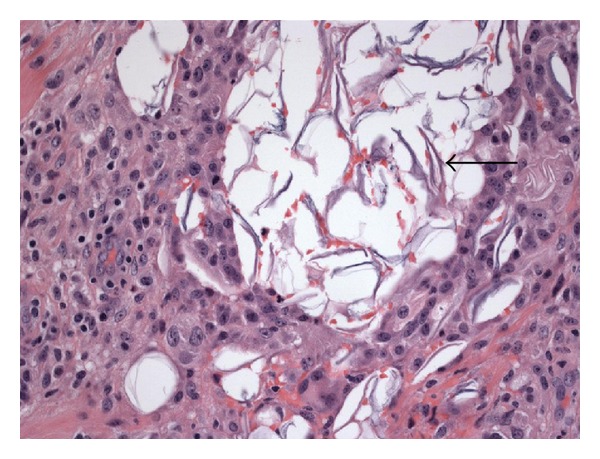
Surrounding the cyst is abundant keratin debris (arrow) admixed with histiocytic and lymphocytic inflammation. Hematoxylin and eosin stain; magnification: ×400.

Subsequent gross inspection of the specimen displayed a focal 1.6 cm cavity surrounded by extensive greenish yellow necrotic-like tissue. By microscopic examination, the cavity corresponded to a squamous inclusion cyst within a lymph node (Figure 3). The cyst was filled with keratin debris, and focal cyst wall showed inflammation. Parts of the cyst were lined by stratified squamous epithelial cells with a prominent granular cell layer and thick laminated hyperkeratotic material (Figure 4). Other parts were lined by multiple layers of polygonal squamous cells without granular cells or hyperkeratosis (Figure 5). The inner cyst surface in these latter parts showed immunopositivity for carcinoembryonic antigen (Figure 6) and Cam 5.2, suggesting a glandular origin of the cyst with extensive squamous metaplasia. However, no apparent glandular structures were seen. All cyst lining cells showed orderly maturation without cytologic atypia. They were negative for estrogen receptor, progesterone receptor, and gross cystic disease fluid protein-15 (GCDFP15). The surrounding tissue exhibited abundant keratin debris admixed with aggregates of histiocytes, foreign body type multinucleated giant cells, abscess formation, and fat necrosis, indicating cyst rupture with extensive reaction (Figure 7). In retrospect, the atypical epithelioid cells on the touch preparation corresponded to reactive histiocytes secondary to cyst rupture. The reactive changes, together with the squamous inclusion cyst, accounted for the 5 cm mass seen clinically and grossly. No malignancy was seen.

The patient recovered uneventfully and has been disease-free for 15 months after surgery.

## 3. Discussion

Spontaneous rupture of a squamous inclusion cyst in the axillary lymph node with focal reaction has been previously described [8, 10]. Extensive reaction secondary to cyst rupture can cause diagnostic confusions. In our case, the histologic changes in the core needle biopsy specimen were consistent with a squamous inclusion cyst in a lymph node, but a metastatic squamous cell carcinoma could not be ruled out. Rapid enlargement of the lesion after core needle biopsy increased the clinical suspicion for malignancy. During intraoperative pathology consultation, numerous single and sheets of atypical epithelioid cells on touch preparation cytology suggested a carcinoma, but inflammation in the background indicated reactive changes. Permanent sections demonstrated a ruptured squamous inclusion cyst within a lymph node with extensive reactive changes. Retrospectively, the atypical epithelioid cells corresponded to aggregates of histiocytes as a reaction to cyst rupture. Confusing histiocytes with carcinoma is a well-known pitfall in cytologic preparations [16]. Our case demonstrated the pre- and intraoperative diagnostic challenges related to a ruptured squamous inclusion cyst following core needle biopsy.

Our previous [10] and current cases suggested squamous metaplasia from a preexisting glandular element as focal cells on the inner cyst surface demonstrated either positive mucicarmine stain or immunopositivity for carcinoembryonic antigen and Cam 5.2—markers of glandular differentiation. The origin of epithelial inclusion cysts in axillary lymph nodes is unclear. Several authors have suggested that epithelial inclusions represent mammary glands [4, 5, 15]. Three mechanisms have been proposed to explain the occurrence of epithelial inclusions. First, an embryologic error may have resulted in the admixture of epithelium and lymphoid tissue. Second, benign mammary epithelium of the breast may have been carried via lymphatic vessels to the lymph nodes. Third, endothelial or other cells within the lymph node may have undergone transformation into an epithelial element. In our case, it was possible that benign mammary glandular tissue was transported through lymphatics to the axillary lymph node during the patient's previous surgery for papilloma in the ipsilateral breast and subsequently underwent squamous metaplasia.

Immunonegativity for GCDFP15, estrogen receptor, and progesterone receptor in the squamous inclusion of our patient does not exclude the possibility of a mammary origin for two reasons. First, GCDFP15 is localized mainly in apocrine metaplastic epithelium of benign breast [17]. Therefore, a squamous epithelial-lined inclusion cyst could still be of mammary origin even if it is negative for GCDFP15. Second, estrogen and progesterone receptors are usually negative in metaplastic squamous epithelium of the breast [18]. Thus their absence in a squamous inclusion cyst does not exclude a mammary origin.

Squamous inclusion cyst of the axillary lymph node should be distinguished from metastatic squamous cell carcinoma, particularly because squamous cell carcinoma may become cystic. While squamous inclusion cyst contains keratin debris, the cystic cavity in squamous cell carcinoma is usually secondary to necrosis and therefore shows necrotic debris [19]. Also, in contrast to squamous inclusion showing orderly cellular maturation and bland cells, squamous cell carcinoma exhibits cellular disorganization and cytologic atypia. The differential diagnosis should also include mucoepidermoid carcinoma. Unlike squamous inclusion cyst that is lined by bland squamous epithelial cells, mucoepidermoid carcinoma often displays an admixture of mucous, squamous, intermediate, and clear cells with variable degrees of cytologic atypia [20]. Furthermore, hyperkeratosis has been reported on the inner cyst surface of most cases of squamous inclusion cysts in the axillary lymph nodes [5, 7, 8, 10] but is rarely seen in mucoepidermoid carcinoma. The benign nature of the cyst in our case was manifested by an orderly maturation of squamous epithelium without cytologic atypia. Our patient has been disease free for 15 months after surgery, further demonstrating the benignity of the lesion.

Our case is the first to show a rapidly enlarging axillary squamous inclusion cyst due to cyst rupture and extensive reaction following core needle biopsy. This case serves to inform the readers to consider a ruptured squamous inclusion cyst in the differential diagnosis if an axillary mass has rapidly enlarged after core needle biopsy.
